# Identification of m^6^A-Associated RNA Binding Proteins Using an Integrative Computational Framework

**DOI:** 10.3389/fgene.2021.625797

**Published:** 2021-03-01

**Authors:** Yiqian Zhang, Michiaki Hamada

**Affiliations:** ^1^Department of Electrical Engineering and Bioscience, Faculty of Science and Engineering, Waseda University, Tokyo, Japan; ^2^AIST-Waseda University Computational Bio Big-Data Open Innovation Laboratory (CBBD-OIL), Tokyo, Japan; ^3^Institute for Medical-Oriented Structural Biology, Waseda University, Tokyo, Japan; ^4^Graduate School of Medicine, Nippon Medical School, Tokyo, Japan

**Keywords:** N6-methyladenosine, RNA binding proteins, RNA modification, enrichment analysis, random forest

## Abstract

N6-methyladenosine (m^6^A) is an abundant modification on mRNA that plays an important role in regulating essential RNA activities. Several wet lab studies have identified some RNA binding proteins (RBPs) that are related to m^6^A's regulation. The objective of this study was to identify potential m^6^A-associated RBPs using an integrative computational framework. The framework was composed of an enrichment analysis and a classification model. Utilizing RBPs' binding data, we analyzed reproducible m^6^A regions from independent studies using this framework. The enrichment analysis identified known m^6^A-associated RBPs including YTH domain-containing proteins; it also identified RBM3 as a potential m^6^A-associated RBP for mouse. Furthermore, a significant correlation for the identified m^6^A-associated RBPs is observed at the protein expression level rather than the gene expression level. On the other hand, a Random Forest classification model was built for the reproducible m^6^A regions using RBPs' binding data. The RBP-based predictor demonstrated not only competitive performance when compared with sequence-based predictions but also reflected m^6^A's action of repelling against RBPs, which suggested that our framework can infer interaction between m^6^A and m^6^A-associated RBPs beyond sequence level when utilizing RBPs' binding data. In conclusion, we designed an integrative computational framework for the identification of known and potential m^6^A-associated RBPs. We hope the analysis will provide more insights on the studies of m^6^A and RNA modifications.

## 1. Introduction

In recent years, RNA modification has emerged as a mode of post-transcriptional gene regulation and has been gaining increasing attention from researchers around the globe. More than 150 types of post-transcriptional modification have been discovered, with N6-methyladenosine (m^6^A) as being one of the most abundant mRNA modification (Roundtree et al., 2017). m^6^A is featured with the DRACH motif(where D = A,G or U;R = A or G;H = A,C or U) and is preferentially located near 3′ untranslated regions (3′ UTR) (Linder et al., 2015). It has been reported that m^6^A participates in essential RNA activities including alternative splicing, export, translation, and decay in the nucleus and cytoplasm (Lee et al., 2020).

M^6^A exerts its function through interaction with several RNA binding proteins that can be considered as m^6^A-associated RBPs. There are three main kinds of known m^6^A-associated RBPs that are also known as m^6^A effectors (Shi et al., 2019), they are writer, eraser, and reader. m^6^A writers are methyltransferases like METTL3, METTL14, WTAP, RBM15/15B, while m6 erasers are demethylases like FTO, ALKBH5, and m^6^A readers are the proteins that can recognize m^6^A like the YTH domain-containing proteins (YTHDF1/2/3), EIF3 (Lee et al., 2020), FMR1 (Edupuganti et al., 2017). These m^6^A effectors cooperate with each other to facilitate both temporal and spatial regulation where writers work in the nucleus to introduce the m^6^A modification which is then recognized by various readers in the nucleus and cytoplasm, which can influence activities of their target RNAs.

Furthermore, the roles of m^6^A and m^6^A-associated RBPs in cancer are being a general interest to researchers. The writer METTL3 was early noticed because of its overexpression in acute myeloid leukemia (AML). It was found that m^6^A promotes the translation of oncogenes like c-MYC, BCL2, and PTEN in the human acute myeloid leukemia MOLM-13 cell line (Vu et al., 2017). Because of necessity of METTL3 in the maintain the leukaemic state, it is identified as a potential therapeutic target for AML (Barbieri et al., 2017). Apart from METTL3, a study found that the reader YTHDF2 silenced in HCC cells can provoke inflammation, vascular reconstruction, and metastatic progression (Hou et al., 2019). Besides, m^6^A and m^6^A reader YTHDF1 have been reported to control anti-tumor immunity. YTHDF1 deficient mice had enhanced therapeutic efficacy of PD-1 checkpoint blockade which suggested YTHDF1's potential in anti-cancer immunotherapy (Han et al., 2019). Therefore, the study of m^6^A and m^6^A-associated RBPs enables us to develop a better understanding of gene regulation mechanism and leads to potential therapeutic opportunities.

To unveil m^6^A's regulation mechanism, it is very necessary to study m^6^A-associated RBPs and their target RNAs. High-throughput sequencing technologies like CLIP-seq (Ule et al., 2005) and RIP-Seq (Zhao et al., 2010) make it feasible to study target RNAs of m^6^A effectors at a transcriptome-wide level. Based on the high-throughput sequencing data, a team developed a database for the collection of these target RNAs (Deng et al., 2020), and another team developed a prediction model focused on the targets of m^6^A readers (Zhen et al., 2020). However, computational resources for identification of m^6^A-associated RBPs are still limited. Though there has been a manually-curated database built for the collection of known m^6^A effectors across species (Nie et al., 2020), to identify potential m^6^A-associated RBPs, it needs to develop efficient computational methods. There are some computational methods that have been used to identify m^6^A-associated RBPs. One such method is to build a prediction model based on deep learning and then extract the sequence features (Zhang and Hamada, 2018; Wang and Wang, 2020). However, not all the RBP motifs are available and sequences can not reflect actual binding status, thus limiting their utility in the identification of m^6^A-associated RBPs. Another group developed an analysis framework to identify cell-specific trans-regulators of m^6^A (An et al., 2020). They identified the association between m^6^A and RBPs but did not take into consideration the interaction such as reading and repelling between them.

In our study we decided to focus on the use of reproducible m^6^A regions for identification of m^6^A-associated RBPs, with consideration of variation among MeRIP-Seq datasets (about 30–60% between studies, even in the same cell type; McIntyre et al., 2020). We aimed to identify m^6^A-associated RBPs from reproducible m^6^A regions using an integrative computational framework. This framework is composed of an enrichment analysis and a classification model. The enrichment analysis allows us to identify RBPs enriched in the m^6^A regions. We were able to identify not only the known m^6^A-associated RBPs like YTH domain-containing proteins, but also a potential m^6^A-associated RBP, RBM3, for mouse. We went on to evaluate the correlation of these m^6^A-associated RBPs with some known m^6^A effectors and compared these to other RBPs. We observed a significant correlation in the protein expression level rather than the gene expression level, which suggested that the m^6^A-associated RBPs participate in potential pathways at the protein-level in gene regulation. On the other hand, we built a Random Forest classification model for the reproducible m^6^A regions using RBPs' binding data in an effort to understand how RBPs contribute to the profiling of m^6^A regions. This RBP-based predictor demonstrated competitive performance when compared with sequence-based methods. Furthermore, the feature importance inferred from this model can be used to reflect m^6^A's action of repelling against RBPs. These results suggested that this framework could enable researchers to infer interaction between m^6^A and m^6^A-associated RBPs beyond sequence level when utilizing RBPs' binding data.

## 2. Materials and Methods

### 2.1. MeRIP-Seq Data Collection and Processing

To obtain MeRIP-Seq data of which cell lines are also available for RPBs' binding data, we manually searched GEO database (Barrett et al., 2013) and finally collected raw MeRIP-Seq FASTA files from four independent studies using human HEK293T cell line (human embryonic kidney 293 cells) from European Nucleotide Archive with accession numbers SRP090687 (Lichinchi et al., 2016), SRP039397 (Schwartz et al., 2014), SRP007335 (Meyer et al., 2012), and SRP162223. We also collected MeRIP-Seq data from four independent studies using mouse embryonic fibroblasts(MEF) with accession numbers SRP039402 (Schwartz et al., 2014), SRP048596 (Geula et al., 2015), SRP115436 (Zhou et al., 2018), and SRP061617 (Zhou et al., 2015).

We pre-processed MeRIP-Seq data by using FastQC (Andrews et al., 2012) for quality control and Cutadapt (Martin, 2011) for adapter-trimming. Then, we used MoAIMS, a transcriptome-based peak-calling tool, to detect m^6^A regions with steps including mapping, keeping uniquely mapped reads, sorting, and marking duplicates (Zhang and Hamada, 2020). MoAIMS is an efficient software we developed based on a statistical framework of a mixture negative-binomial distribution. We run MoAIMS with default parameters except that we set sep_bin_info=F when analyzing studies with replicates. MoAIMS called enriched regions at 200-bp resolution as default, therefore we obtained m^6^A-enriched regions with a size of 200 bp for each MeRIP-Seq sample, and then we identified reproducible m^6^A regions using the criteria that regions are called in at least 60% of the replicates in any one study and further in at least three studies.

### 2.2. The Enrichment Analysis

We retrieved binding site data of RBPs from the POSTAR2 database (Zhu et al., 2019) and identified RBPs enriched in the reproducible m^6^A regions. A permutation test was adopted to assess the significance of RBP's binding in the m^6^A regions. The rest of regions in genes with m^6^A was used as control and then sampled 1,000 times. We kept the ratio of the number of bins in exons to the number of bins spanning exons the same for both m^6^A and control regions to avoid the regions' position being a confounding factor. For each RBP, we calculated the enrichment ratio using the Equation (1) where *N*_*t*_ is the number of m^6^A regions with the RBP and *E*(*N*_*c*_) is the average number of control regions with the RBP from 1,000 times of sampling. Then, a *p*-value was calculated as the proportion of *N*_*c*_ which were equal to or greater than *N*_*t*_. After that, multiple testing was performed using Benjamini and Hochberg (1995).

(1) $$\begin{array}{r} R=\frac{N_{t}}{E\left(N_{c}\right)} \end{array}$$

### 2.3. The Classification Model

We built a Random Forest (RF) classifier to evaluate how much RBPs contribute in discriminating reproducible m^6^A regions. We used the human m^6^A regions with RBPs' binding as the positive data (13,978 in total) and generated 10 sets of control data from the control regions which were set to be an equal data size. We kept the ratio of the number of bins in exons to the number of bins spanning exons the same in both m^6^A and control regions. The binding information (1 for binding, 0 for non-binding) of RBPs was used as the input features. The data was divided into training and test groups at a ratio of 80:20. We implemented the RF classifier using the R package caret (Kuhn, 2008) and randomForest (Liaw and Wiener, 2002) with 5-fold cross validations and “mtry” (the tuning parameters) as 8 (nearly the square root of the number of features). We used the accuracy to measure the performance of the models as shown in the Equation (2) where TP is true positive, TN is true negative, FP is false positive and FN is false negative.

(2) $$\begin{array}{r} Accuracy=\frac{TP+TN}{TP+TN+FP+FN} \end{array}$$

**Figure 1:**
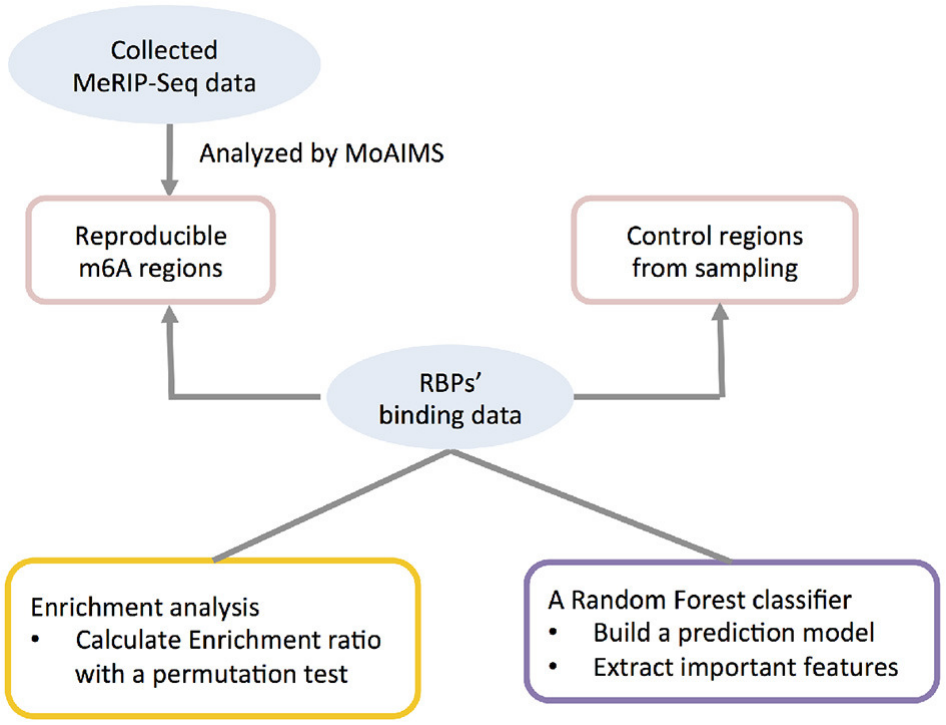
Illustration of the integrative computational framework.

A analysis framework including the procedures above was summarized in Figure 1.

## 3. Results

### 3.1. Identification of m^6^A-Associated RBPs Enriched in Reproducible m^6^A Regions

Because of the considerable variation in the m^6^A datasets (McIntyre et al., 2020), we generated *reproducible* m^6^A regions by collecting MeRIP-Seq data from nine samples of human HEK293T cell line of four independent studies and six samples of mouse MEF cell line of four independent studies. The details of detection of these m^6^A regions are provided in section 2. With a relatively strict criteria, we finally obtained 14,803 reproducible m^6^A regions for HEK293T cell line and 5,576 reproducible m^6^A regions for MEF cell line.

To identify RBPs enriched in m^6^A regions, 71 RBPs for HEK293T/HEK293 and nine RBPs for MEF were retrieved from the POSTAR2 database. For each RBP, we calculated an enrichment score and assessed its significance using a permutation test as described in section 2. When setting the threshold for the enrichment ratio to ≥1.3 and FDR (false discovery rate) adjusted *p*-value to ≤0.05, we obtained enriched RBPs listed in Table 1. For HEK293T, we identified several known m^6^A readers including YTH family proteins, FMR1, EIF3, and m^6^A writers RBM15/15B, a component of the WTAP-METTL3 complex (Patil et al., 2016; Lee et al., 2020). For MEF, we found a common RBP, CPSF6, which is enriched for both human and mouse. CPSF6 is a polyadenylation cleavage factor and has been reported to be associated with VIRMA, which mediates preferential m^6^A methylation in the 3' UTR and near stop codon and participates alternative polyadenylation (APA) in human (Yue et al., 2018). Another study found YTHDC1's association with CPSF6 during mouse oocyte development (Kasowitz et al., 2018). In addition, we noticed that RBM3 was highly enriched in m^6^A regions of MEF. RBM3 is an important regulator of circadian gene expression by controlling APA (Liu et al., 2013), therefore we suggest that RBM3 could be associated with m^6^A in the APA regulation process. The full list of enrichment ratios for each of the RBPs is provided in Supplementary Tables 1, 2. Besides, for each enriched RBP (overlap with more than 100 m^6^A regions), we also listed the RBPs that more than 60% of the enriched RPB is overlapped with for HEK293T in Supplementary Table 3. As expected, YTHDF1 and DDX3X were shown to have the highest overlapping percentage as they have a considerable overlap with m^6^A regions.

**Table 1:** RNA binding proteins (RBPs) enriched in reproducible m^6^A regions.

**HEK293T**	Enrichment ratios^*^	**# m^**6**^A regions with RBPs**	*p*-value^**^	**FDR adjusted *p*-value**
YTHDF2	3.90	6,964	<0.001	<0.003
RBM15	2.73	3,534	<0.001	<0.003
YTHDF3	2.70	52	<0.001	<0.003
YTHDF1	2.49	9,196	<0.001	<0.003
RBM15B	2.32	6,375	<0.001	<0.003
YTHDC1	2.15	7,224	<0.001	<0.003
EIF3D	1.88	593	<0.001	<0.003
NOP58	1.74	159	<0.001	<0.003
HNRNPH1	1.57	47	0.002	0.006
NUDT21	1.48	5,201	<0.001	<0.003
FMR1	1.46	4,443	<0.001	<0.003
DDX3X	1.44	9,470	<0.001	<0.003
EIF3A	1.39	293	<0.001	<0.003
CPSF6	1.34	3,593	<0.001	<0.003
CPSF7	1.31	4,413	<0.001	<0.003
**MEF**	Enrichment ratio^*^	**# m**^**6**^**A regions with RBPs**	***p***-value^**^	**FDR adjusted** ***p*****-value**
RBM3	5.81	485	<0.001	<0.001
CREBBP	2.47	24	<0.001	<0.001
SRSF2	2.24	793	<0.001	<0.001
SRSF1	2.13	467	<0.001	<0.001
CPSF6	2.07	94	<0.001	<0.001
CIRBP	1.76	401	<0.001	<0.001

* *RBPs are ranked by their enrichment ratios*.

** *P-values were calculated from 1,000 times of permutation. When p-value is zero, it is shown in the table as < 0.001 because it is possible that the p-value is actually < 0.001 if times of permutation were increased*.

**Figure 2:**
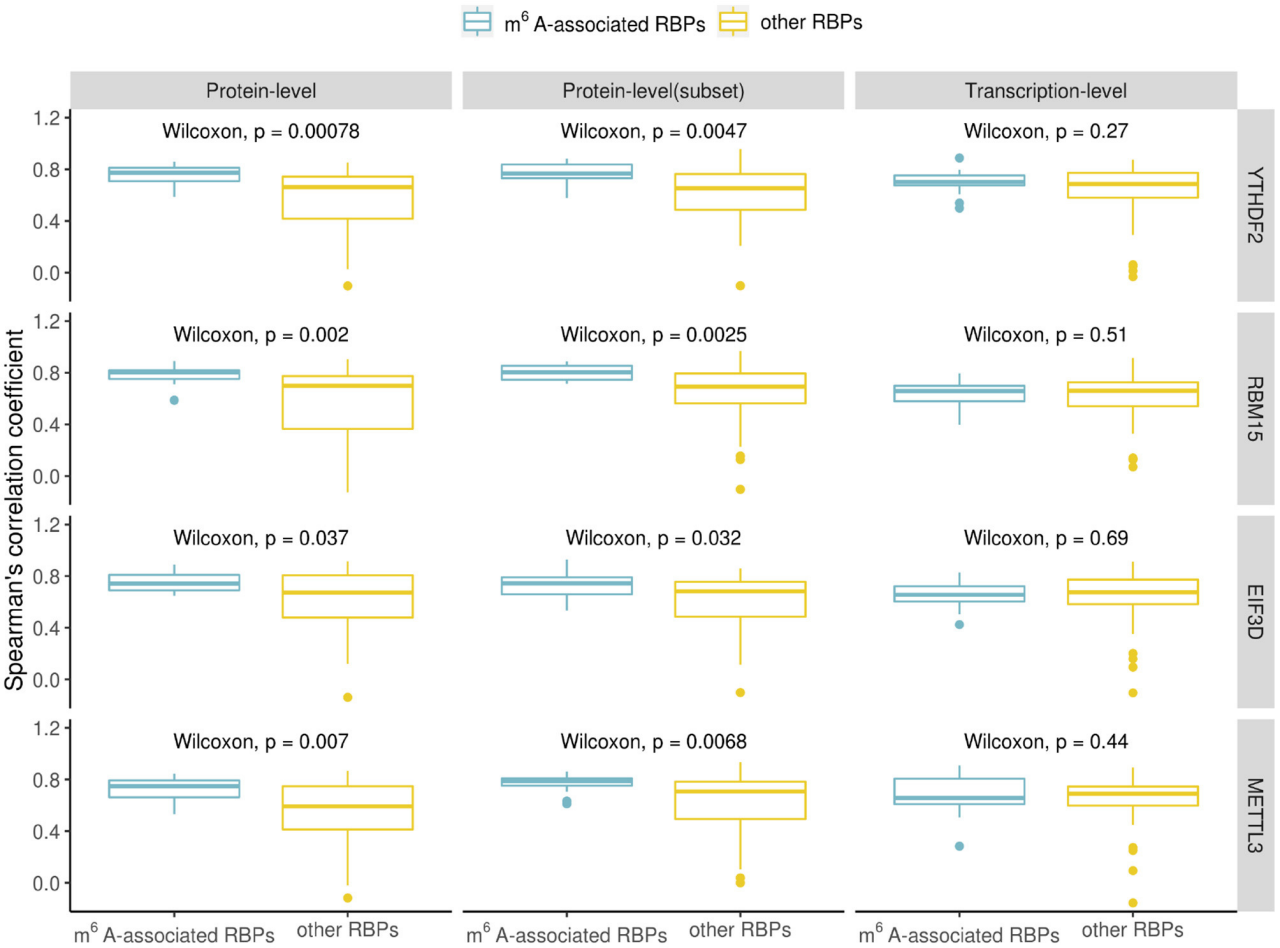
Comparison of the correlation values for known m^6^A effectors (YTHDF2, RBM15, EIF3D, and METTL3) with identified m^6^A-associated RBPs (15 in total) or other RBPs. The boxplot shows the distribution of the Spearman's correlation coefficient between known m^6^A effectors and identified m^6^A-associated RBPs/other RBPs at the protein- and transcript-level (the subset protein-level results describe the correlation coefficients calculated from a subset of the protein data which included only 17 adult tissues). Significance was evaluated using a one-sided Wilcoxon test.

**Figure 3:**
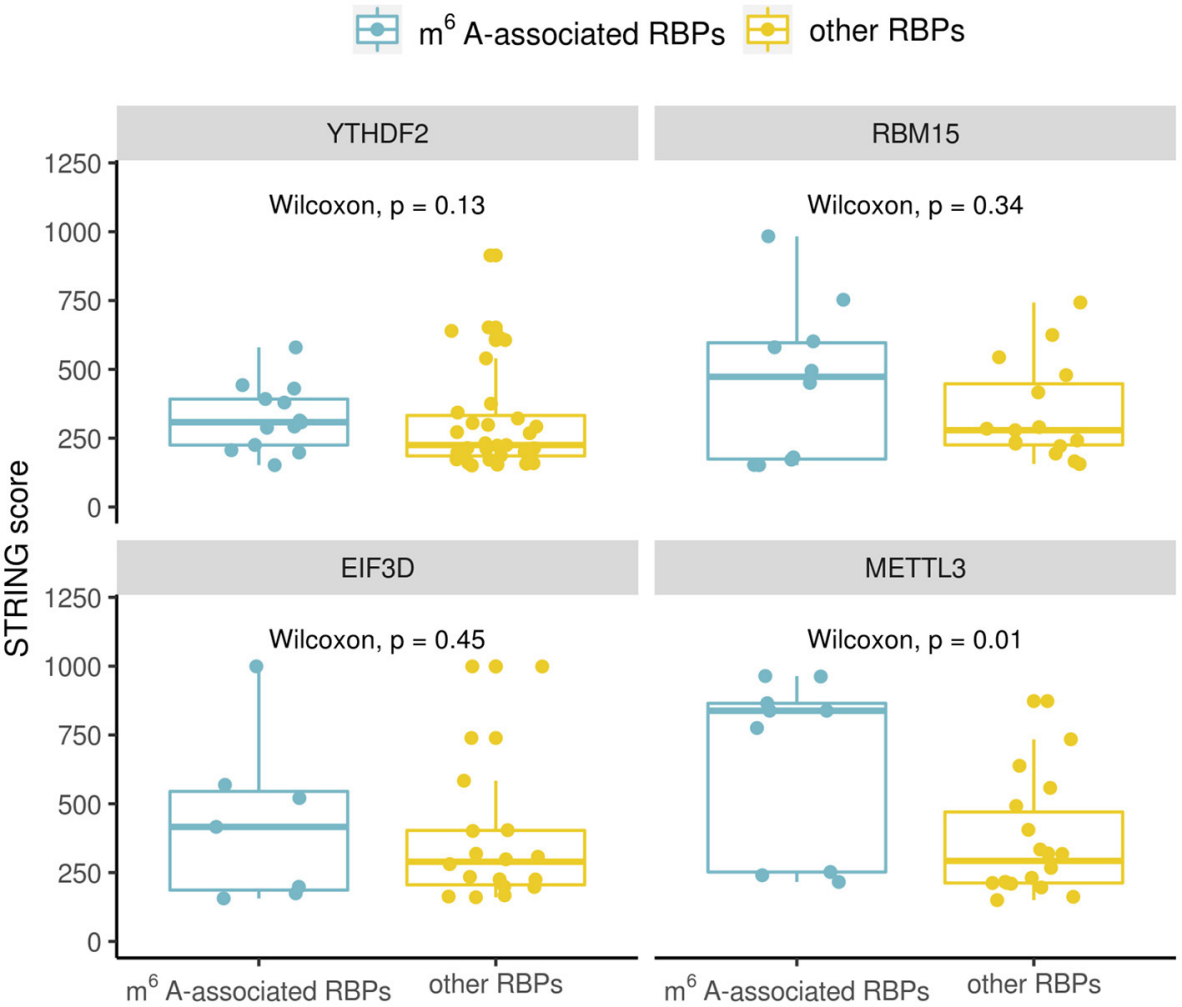
Comparison of protein-protein interactions between known m^6^A effectors (YTHDF2, RBM15, EIF3D, and METTL3) and identified m^6^A-associated RBPs (15 in total)/other RBPs. The boxplot shows the distribution of the interaction scores between known m^6^A effectors and identified m^6^A-associated RBPs/other RBPs. Significance was evaluated using a one-sided Wilcoxon test.

The RBPs in Table 1 are considered as m^6^A-associated RBPs, therefore we wondered how they are correlated with known m^6^A effectors when compared with other RPBs at both the transcription and the protein expression level. We performed a correlation analysis for all the human RBPs. To do the correlation analysis at the transcription level, we downloaded Illumina Body Map (HBM) (Asmann et al., 2012; Barbosa-Morais et al., 2012; Derrien et al., 2012) from ArrayExpress (Athar et al., 2019) with the accession number E-MTAB-513, which provides gene expression data for 16 human tissues. For the correlation analysis at the protein level, we downloaded mass spectrometry data from Human Proteome Map (HPM) (Kim et al., 2014) for 30 human tissues/cell lines. We checked some known m^6^A effectors including YTHDF2, RBM15, EIF3D which ranked at the top of Table 1 and METTL3 of which binding data is not available but is a well-known m^6^A writer, and compared their correlation with the identified m^6^A-associated RBPs (15 in total) or with the rest of RBPs (56 in total). Correlation was calculated using the Spearman's correlation coefficient. We observed a similar trend in all the investigated known m^6^A effectors which showed that the identified m^6^A- associated RBPs are more correlated with them at the protein-level than the transcription level (Figure 2). Because the protein data included more tissues/cell lines than the transcription data, we chose to compare a subset of 17 adult tissues to check the correlation values for avoiding any biased introduced by different dataset sizes. The higher correlation at the protein level was still observed in this subset evaluation as shown in Figure 2. Some studies have reported cases of gene regulation with dependency between m^6^A-associated RBPs such as METTL3 and YTHDF2 (Chen et al., 2018; Kasowitz et al., 2018). This observation supports the hypothesis that m^6^A-associated RBPs are more likely to participate in potential pathways at the protein level. Then, we went on to confirm to if these higher correlation values are the result of protein-protein interactions. To do this we retrieved the protein-protein interaction data from STRING (von Mering et al., 2005). The available interaction scores do not show significant difference between m^6^A-associated RBPs and other RBPs except for METTL3 (Figure 3). Because the protein-protein interaction data is still limited, from the available data it is suggested that the higher correlation at the protein-level is marginally related to protein-protein interaction. m^6^A modification is a dynamic process involving both temporal and spatial regulation between m^6^A effectors, therefore it is expected to have further studies to unveil the regulation mechanism of these proteins.

### 3.2. Identification of m^6^A-Associated RBPs Contributing to the Classification of m^6^A Regions

**Figure 4:**
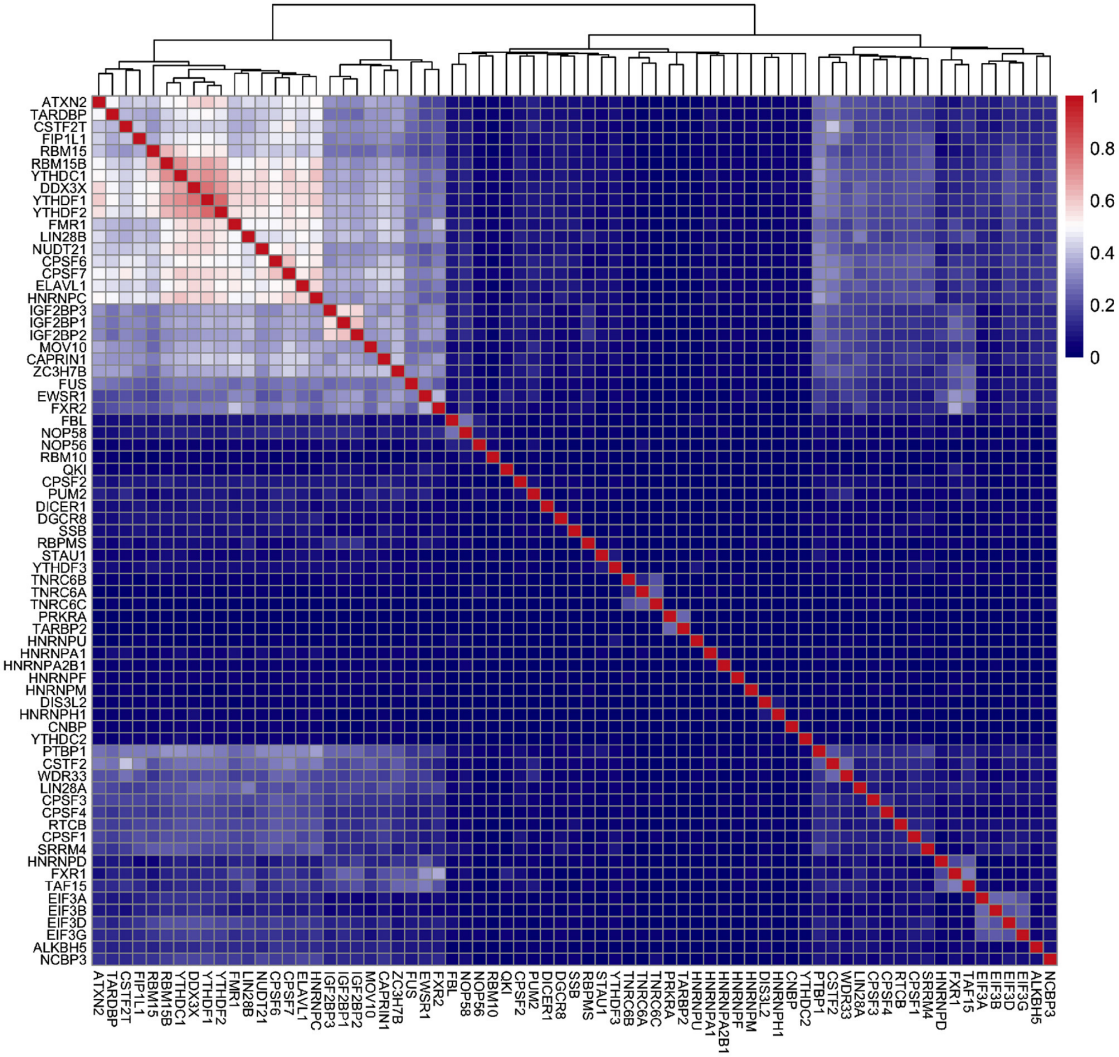
Clustering of RNA binding proteins (RBPs) in the m^6^A regions of HEK293T cell line. X-axis and Y-axis represent the names of the RBPs. The color scale indicates the cosine similarity between the RBPs.

**Figure 5:**
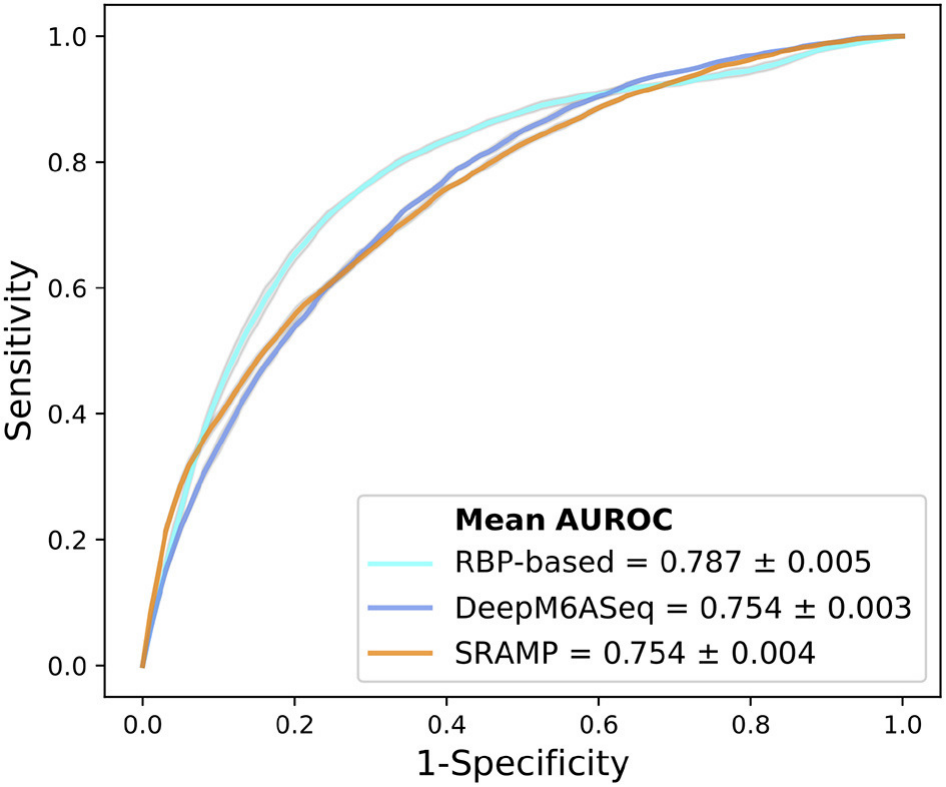
Comparison of AUROC between the RBPs (RNA binding proteins)-based predictor, DeepM6ASeq, and SRAMP in mature mRNA mode for the classification of HEK293T m^6^A regions. The plot represents average ROC from ten times of sampling control regions for each predictor.

**Figure 6:**
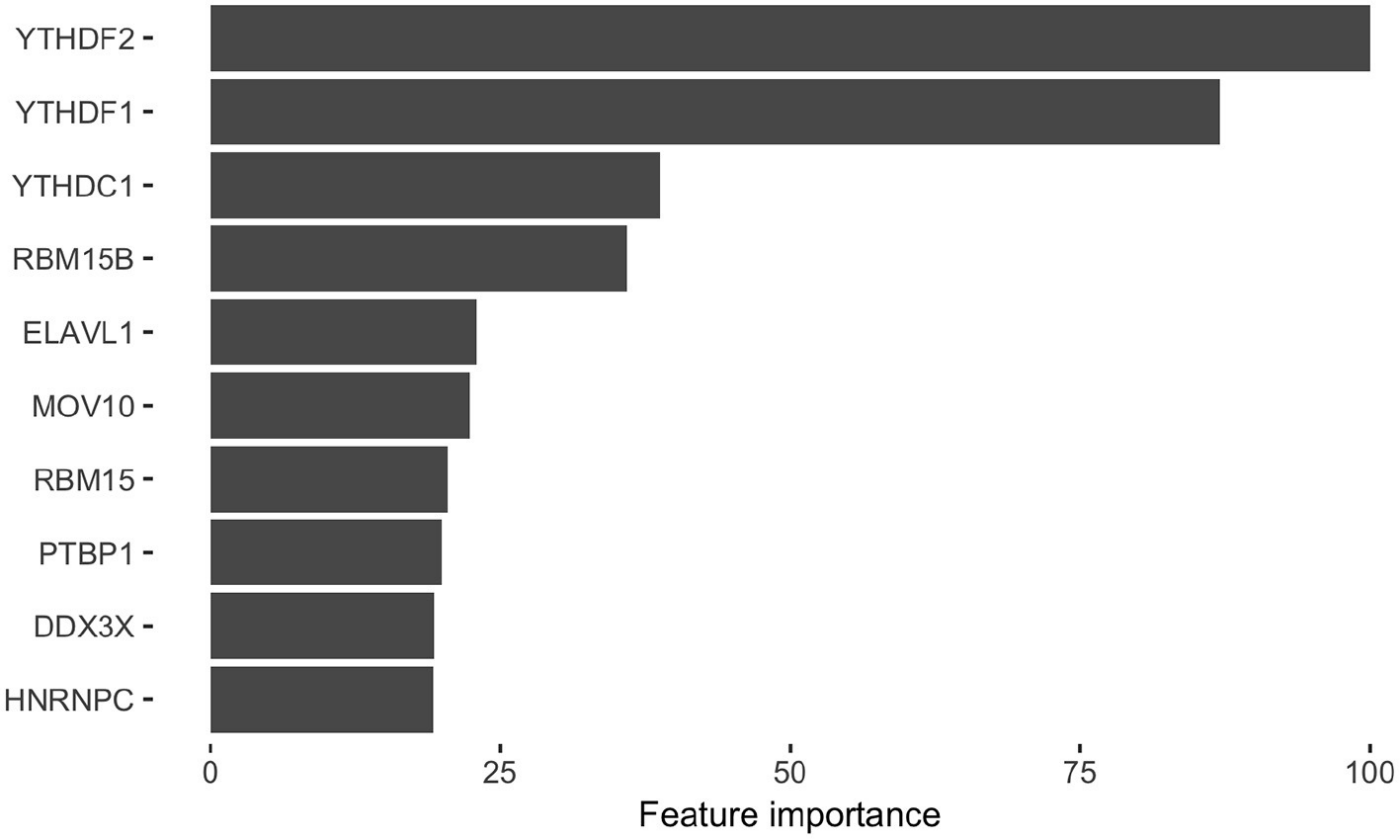
Top 10 RNA binding proteins (RBPs) identified from the classification of the HEK293T reproducible m^6^A regions. The bar graph shows the top 10 RBPs extracted from the classifier for the m^6^A regions. X-axis represents the name of RBPs and Y-axis represents the average importance score from ten times of sampling control regions.

After we identified RBPs enriched in the reproducible m^6^A regions, we wanted to develop a more comprehensive understanding of how RBPs' binding contributes to the profile of m^6^A regions. To do this, we performed a further analysis on the human RBPs. First, we investigated the overall profile of the binding information of RBPs (0 for non-binding and 1 for binding) in the reproducible m^6^A regions. We calculated the pairwise distance between RBPs using cosine similarity and performed clustering (Figure 4). The result of the clustering analysis demonstrated the co-occurrence of YTH family proteins and RBM15B which all ranked in the top of the enrichment analysis. Then, we built a Random Forest classifier which incorporated the binding information for each of the RBPs as features. The details of models are described in section 2. The classifier achieved an average accuracy of 0.736 and AUROC (Area Under Receiver Operating Characteristic) of 0.788 as shown in Figure 5. We also compared the RBP-based classifier with two sequence-based predictors SRAMP (Zhou et al., 2016) in mature mRNA mode and DeepM6ASeq which showed an accuracy of 0.660 and 0.686, respectively and AUROC of 0.754 for both (Figure 5). We plotted top 10 most important features as shown in Figure 6 and among them found the enriched m^6^A-associated RPBs such as the readers YTHDF1/2, YTHDC1, the writers RBM15/15B. Besides, it is noticed that ELAVL1 also has contribution to the classification of m^6^A regions to some extent. ELAVL1 is reported to have action of being repelled by m^6^A in general that can lead to RNA decay (Wang et al., 2014; Lee et al., 2020). The repelling action of m^6^A against ELAVL1 is consistent with the enrichment results, which show that its enrichment ratio is 0.816. In summary, the RBP-based classifier not only demonstrated competitive performance in the prediction of reproducible m^6^A regions but also helped to infer interaction between m^6^A and m^6^A-associated RBPs beyond sequence level when combined with the results of the enrichment analysis.

## 4. Discussion

Utilizing the binding information of RBPs, this computational framework enabled us to identify potential m^6^A-associated RBPs and infer their interaction with m^6^A. This analysis serves as a first step, and future analyses may include some improvements and expansions. First, this framework was designed and tested on a limited number of cell types and organisms. With the increasing amount of data available for m^6^A and RBPs in more cell lines and tissues, this framework could be tested on much larger datasets and may provide valuable insights into the m^6^A regulatory network. Especially, this framework is promising in the application of cancer research. Several studies have identified function of m^6^A effectors like METTL3/14, YTHDF1/2, and IGF2BP1 in multiple cancer types (Cui et al., 2017; Li et al., 2018; Chen et al., 2019; Han et al., 2019; Müller et al., 2019). This framework is expected to provide clues for potential m^6^A effectors and the interaction among them in cancer research. In addition, this framework could be applied to other RNA modifications such as N1-methyladenosine (m1A) (Dominissini et al., 2016), and 5-methylcytidine(m5C) (Amort et al., 2017), which have also been identified as critical RNA modification. Such analyses could help improve experimental design in wet lab applications and help researchers narrow their focus. Third, apart from RBPs, other genomic features like transcription factors and histone modification are worth inspecting for studying the m^6^A regulation networks at multiple layers. These applications highlight the future utility of this framework and its value in the current research climate.

## 5. Conclusion

We designed an integrative computational framework for identification of m^6^A-associated RBPs in reproducible m^6^A regions. This computational framework is composed of an enrichment analysis and a classification model. Using the enrichment analysis, we were able to identify known m^6^A-associated RBPs and several potential ones including RBM3 from mouse. These identified m^6^A-associated RBPs show a significant degree of correlation at their protein level, although this is not seen in their transcriptional profile, which suggests that these m^6^A-associated RBPs participate in potential pathways at the protein-level in gene regulation. On the other hand, we built a classification model for m^6^A regions using a Random Forest algorithm that uses RBPs' binding information as its input features. The RBP-based predictor not only demonstrated comparable performance to sequence-based predictions but also helped infer interaction between m^6^A and m^6^A-associated RBPs like actions of reading and repelling beyond sequence level. We hope that this analysis framework can assist biologists in their study of RNA modifications.

## Data Availability Statement

The datasets presented in this study can be found in online repositories. The names of the repository/repositories and accession number(s) can be found in the article/Supplementary Material.

## Author Contributions

YZ conceived this study, implemented the methods, performed the experiments, and wrote the draft. MH supervised this study and revised the manuscript critically. YZ and MH contributed to the construction of methods and analysis/interpretation of the data. All authors read and approved the final manuscript.

## Conflict of Interest

The authors declare that the research was conducted in the absence of any commercial or financial relationships that could be construed as a potential conflict of interest.
