# Supplementary material for: Identification of m6A-Associated RNA Binding Proteins Using an Integrative Computational Framework
Source: Front Genet. 2021 Mar 1;12:625797. doi: 10.3389/fgene.2021.625797 (PMC7957075; doi:10.3389/fgene.2021.625797)
Supplement: Supplementary file 1 [file Data_Sheet_1.PDF]

Supplementary Material

1 SUPPLEMENTARY TABLES

1.1 Tables

Table S1: Enrichment ratios for RNA binding proteins (RBPs) associated with the reproducible m^{6}A regions identified in the human HEK293T dataset

HEK293T	Enrichment ratio	# m^{6}A regions with RBP	p-value*	FDR adjusted p-value
ALKBH5	1.09	265	0.065	0.171
ATXN2	1.04	5378	<0.001	<0.003
CAPRIN1	1.17	2331	<0.001	<0.003
CNBP	1.34	32	0.052	0.142
CPSF1	0.96	558	0.850	1.000
CPSF2	1.09	124	0.157	0.384
CPSF3	0.93	680	0.988	1.000
CPSF4	0.89	506	1.000	1.000
CPSF6	1.34	3593	<0.001	<0.003
CPSF7	1.31	4413	<0.001	<0.003
CSTF2	0.68	1104	1.000	1.000
CSTF2T	0.86	2738	1.000	1.000
DDX3X	1.44	9470	<0.001	<0.003
DGCR8	1.07	67	0.273	0.606
DICER1	0.94	92	0.773	1.000
DIS3L2	0.46	11	1.000	1.000
EIF3A	1.39	293	<0.001	<0.003
EIF3B	1.10	255	0.073	0.185
EIF3D	1.88	593	<0.001	<0.003
EIF3G	1.14	453	0.001	0.003
ELAVL1	0.82	4594	1.000	1.000
EWSR1	0.75	853	1.000	1.000
FBL	1.22	88	0.026	0.074
FIP1L1	1.16	3419	<0.001	<0.003
FMR1	1.46	4443	<0.001	<0.003
FUS	0.79	1179	1.000	1.000
FXR1	0.95	178	0.795	1.000
FXR2	1.23	966	<0.001	<0.003
HNRNPA1	0.60	53	1.000	1.000
HNRNPA2B1	0.67	11	0.948	1.000
HNRNPC	0.95	5404	1.000	1.000
HNRNPD	0.29	123	1.000	1.000
HNRNPF	0.95	35	0.661	1.000

Continued on next page

Table S1 – *Continued from previous page*

HEK293T	Enrichment ratio	# m^6A regions with RBP	p-value*	FDR adjusted p-value
HNRNPH1	1.57	47	0.002	0.006
HNRNPM	0.34	7	1.000	1.000
HNRNPU	0.31	2	0.996	1.000
IGF2BP1	1.07	1847	<0.001	<0.003
IGF2BP2	0.91	1583	1.000	1.000
IGF2BP3	0.77	1796	1.000	1.000
LIN28A	1.01	791	0.426	0.864
LIN28B	1.06	4997	<0.001	<0.003
MOV10	0.60	2001	1.000	1.000
NCBP3	1.29	529	<0.001	<0.003
NOP56	0.82	20	0.860	1.000
NOP58	1.74	159	<0.001	<0.003
NUDT21	1.48	5201	<0.001	<0.003
PRKRA	1.65	5	0.176	0.417
PTBP1	0.61	1397	1.000	1.000
PUM2	0.41	108	1.000	1.000
QKI	0.48	32	1.000	1.000
RBM10	1.20	17	0.251	0.575
RBM15	2.73	3534	<0.001	<0.003
RBM15B	2.32	6375	<0.001	<0.003
RBPMS	0.68	87	0.999	1.000
RTCB	1.17	619	<0.001	<0.003
SRRM4	0.95	490	0.908	1.000
SSB	1.06	73	0.319	0.686
STAU1	0.23	43	1.000	1.000
TAF15	0.76	239	1.000	1.000
TARBP2	0.66	3	0.848	1.000
TARDBP	0.90	4194	1.000	1.000
TNRC6A	1.05	9	0.478	0.943
TNRC6B	1.18	11	0.338	0.706
TNRC6C	0.38	2	0.981	1.000
WDR33	0.58	650	1.000	1.000
YTHDC1	2.15	7224	<0.001	<0.003
YTHDC2	0.96	36	0.636	1.000
YTHDF1	2.49	9196	<0.001	<0.003
YTHDF2	3.90	6964	<0.001	<0.003
YTHDF3	2.70	52	<0.001	<0.003
ZC3H7B	0.62	1889	1.000	1.000

* P-values were calculated from 1000 times of permutation. When p-value is zero, it is shown in the table as < 0.001 becasue it is possible that the p-value is actually less than 0.001 if times of permutation were increased.

Table S2: Enrichment ratios for RNA binding proteins (RBPs) in the reproducible m^{6}A regions identified in the mouse MEF dataset

MEF	Enrichment ratio	# m^{6}A regions with RBP	p-value*	FDR adjusted p-value
CIRBP	1.76	401	<0.001	<0.001
CPSF6	2.07	94	<0.001	<0.001
CREBBP	2.47	24	<0.001	<0.001
MBNL1	2.16	8	0.040	0.045
MBNL2	1.55	5	0.216	0.216
MBNL3	NA	1	NA	NA
RBM3	5.81	485	<0.001	<0.001
SRSF1	2.13	467	<0.001	<0.001
SRSF2	2.24	793	<0.001	<0.001

* P-values were calculated from 1000 times of permutation. When p-value is zero, it is shown in the table as < 0.001 becasue it is possible that the p-value is actually less than 0.001 if times of permutation were increased.

Table S3. Overlapped RNA binding proteins (RBPs) for proteins enriched in the human HEK293T dataset

RBPs enriched in m^6A	RBPs with overlapping ratio more than 60%*
YTHDF2	YTHDF1(89.7%), DDX3X(80.5%), YTHDC1(68.5%), RBM15B(60.6%)
RBM15	YTHDF1(86.8%), RBM15B(83.6%), DDX3X(82.8%), YTHDC1(81.7%), YTHDF2(74.6%)
YTHDF1	DDX3X(77.4%), YTHDF2(67.9%), YTHDC1(63.0%)
RBM15B	YTHDF1(80.4%), DDX3X(76.9%), YTHDC1(75.7%), YTHDF2(66.2%)
YTHDC1	YTHDF1(80.2%), DDX3X(76.3%), RBM15B(66.8%), YTHDF2(66.0%)
EIF3D	DDX3X(87.0%), YTHDF1(83.1%), YTHDC1(72.0%), YTHDF2(68.3%), RBM15B(65.6%)
NOP58	DDX3X(81.1%), YTHDC1(77.4%), YTHDF1(75.5%), RBM15B(74.2%), CPSF7(73.0%), NUDT21(71.1%), HNRNPC(70.4%), LIN28B(66.7%), YTHDF2(63.9%), FMR1(62.3%), CPSF6(60.4%)
NUDT21	DDX3X(76.7%), YTHDF1(75.7%), YTHDC1(67.7%), YTHDF2(60.5%)
FMR1	DDX3X(87.2%), YTHDF1(83.1%), YTHDF2(69.5%), YTHDC1 (69.1%)
DDX3X	YTHDF1(75.1%)
EIF3A	DDX3X(85.0%), YTHDF1(80.9%), YTHDC1(70.3%), YTHDF2(68.9%), RBM15B(68.6%)
CPSF6	DDX3X(84.5%), YTHDF1(82.0%), YTHDC1(72.6%), YTHDF2(68.7%), CPSF7(67.2%), HNRNPC(65.0%), RBM15B(63.9%), NUDT21(61.9%)
CPSF7	DDX3X(83.3%), YTHDF1(82.4%), YTHDC1(75.0%), YTHDF2(68.5%), HNRNPC(64.4%), RBM15B(63.4%), NUDT21(60.5%)

*Numbers in brackets indicate the percentage of overlap and are listed in decreasing order.
